# Supplementary material for: Evaluation of the Toxic Activity of the Graphene Oxide in the Ex Vivo Model of Human PBMC Infection with Mycobacterium tuberculosis
Source: Microorganisms. 2023 Feb 22;11(3):554. doi: 10.3390/microorganisms11030554 (PMC10059016; doi:10.3390/microorganisms11030554)

Supplementary Figure S1. Representative atomic force microscopy image of a single GO sheet from the diluted water dispersion and the relative height profile. GO sheets. Sample has a hydrodynamic radius ranging from 500 to 600 nm and an average height of 1nm, indicating single-layered GO sheets.

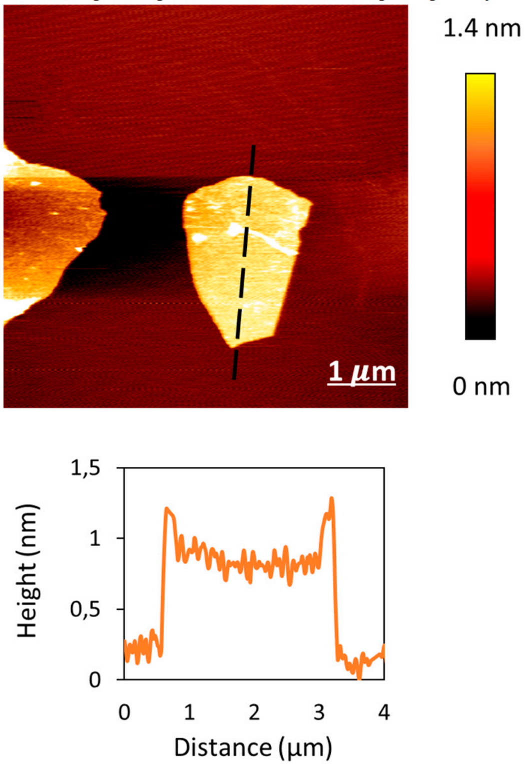

Supplementary Figure S2. GO auto-fluorescence and GO-7AAD complexation evaluated through cytofluorimetric analysis. RPMI cell culture medium added with GO 10 (C,D and I, J) and 100 µg/ml (E, F and K, L). RPMI without GO was used as control (A, B and G, H) and then incubated with or without 7AAD. Samples incubated without 7AAD (A-F) were analysed through Side Scatter versus Forward Scatter gating, and then through PC5-5-A versus Side Scatter gating. Sample incubated with 7AAD (G-L) were analysed through Side Scatter versus Forward Scatter gating, and then through 7AAD PC5-5-A versus Side Scatter gating.

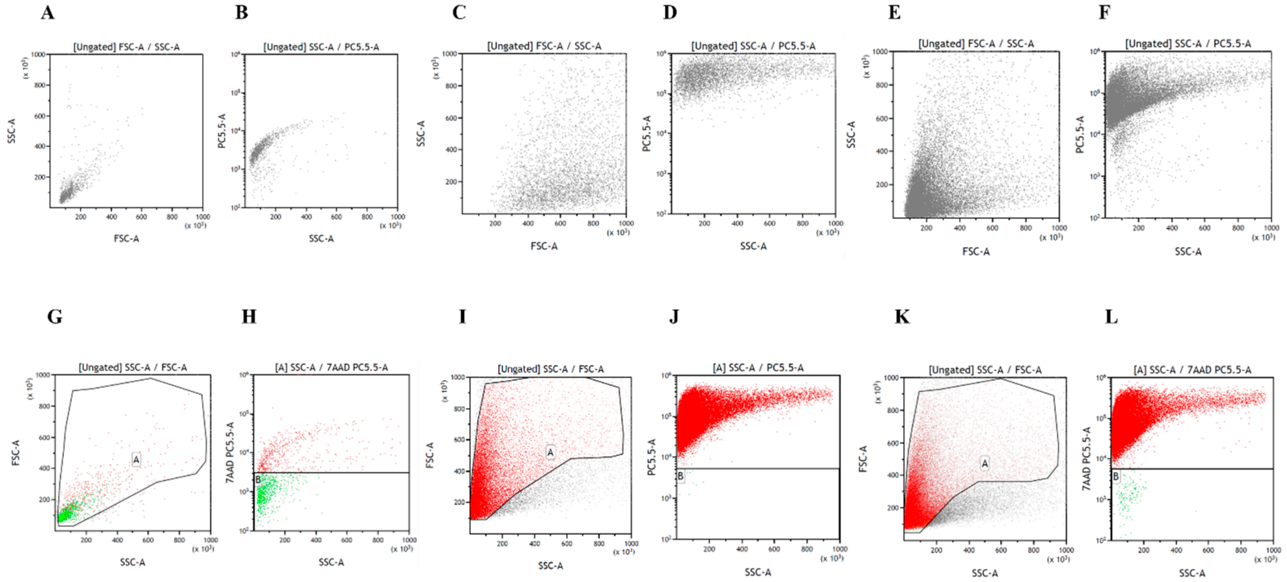

Supplement: Supplementary file 1 [file microorganisms-11-00554-s001.zip › microorganisms-2188143-supplementary.pdf]
